# Revisiting Rosai Dorfman disease: A rare histiocytic disorder with nodal and extranodal involvement

**DOI:** 10.4102/sajr.v27i1.2729

**Published:** 2023-10-30

**Authors:** Smily Sharma, Poonam Sherwani, Venkata S. Arunachalam, Rahul Dev

**Affiliations:** 1 Department of Diagnostic and Interventional Radiology, All India Institutes of Medical Sciences (AIIMS) Jodhpur, Jodhpur, India; 2 Department of Radiodiagnosis, All India Institutes of Medical Sciences (AIIMS) Rishikesh, Rishikesh, India; 3 Department of Imaging Sciences and Interventional Radiology, Sree Chitra Tirunal Institute for Medical Sciences and Technology, Thiruvananthapuram, India

**Keywords:** Rosai Dorfman disease, emperipolesis, massive lymphadenopathy, sinus histiocytosis, histiocytosis

## Abstract

Rosai Dorfman disease is a rare but benign and self-limiting disorder. A case of Rosai Dorfman disease in a 16-year-old female with classic radiological and pathological findings is presented. T2W hypointense signal of the soft tissue masses was a distinguishing MRI feature.

**Contribution:**

Rosai Dorfman disease is often overlooked in the differentials of lymphadenopathy and soft tissue masses on account of its rarity. This case report comprehensively discusses the imaging approach to this rare disorder.

## Introduction

Rosai Dorfman disease (RDD) is a rare histiocytic disorder characterised by the proliferation of macrophages. It commonly presents as massive lymphadenopathy in children and adolescents. However, extranodal manifestations involving virtually any organ of the body may be seen in approximately half of the patients. Nevertheless, it remains a benign and self-limiting disease with management focused mainly on removing any compressive effects of massive lymphadenopathy.^1^ This report describes a case of an adolescent female who presented with eyelid, cheek, neck and right thigh swellings and was diagnosed as a typical case of RDD with nodal and extranodal involvement.

## Patient presentation

A 16-year-old female presented to the Otorhinolaryngology department with multiple cheek and neck swellings over 9 years. The swellings were insidious in onset and progressive in nature, with subsequent bilateral upper eyelid swelling. She had a focal swelling in her left thigh for 2 years. She also had recurrent episodes of nasal obstruction for about 7 years. There was no associated pain, fever or weight loss present. No history of any immune compromise, prior medical event or similar findings in family members was noted.

On examination, the patient had smooth, firm swellings involving both eyelids with normal vision and no limitation of eye movements. Diffuse enlargement of both parotid and submandibular glands was noted along with cervical lymphadenopathy. The swellings were not associated with tenderness, redness or heat. The patient had a normal complete blood count with a mildly raised erythrocyte sedimentation rate (ESR). Lactate dehydrogenase (LDH) levels and viral markers were within normal limits.

**FIGURE 1:**
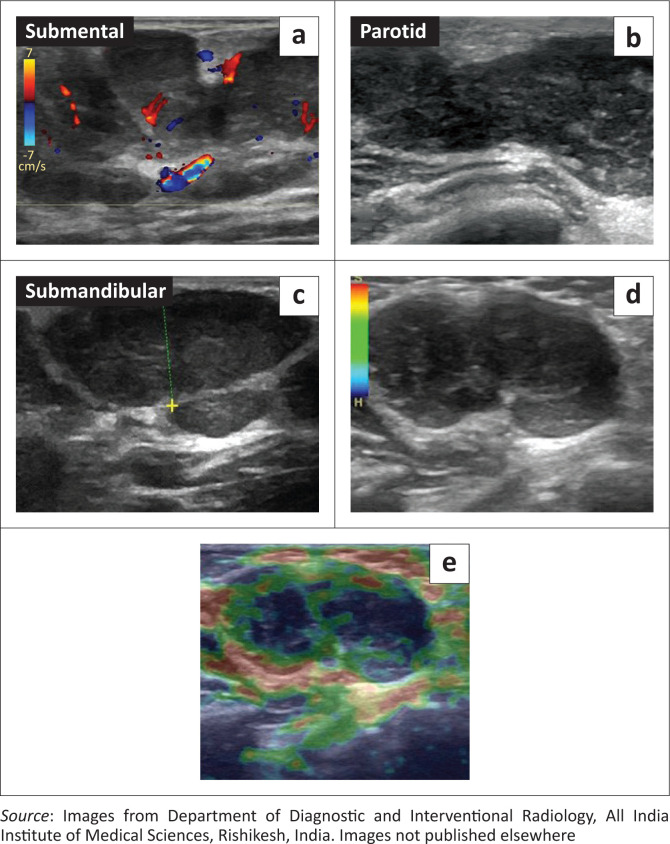
Colour doppler (a) and grey scale (b, c) ultrasound images show enlarged hypoechoic lymph nodes with increased vascularity (a) in the submental, parotid and submandibular stations. (d, e) Ultrasound elastography of an enlarged lymph node shows an intermediate elasticity score.

**FIGURE 2:**
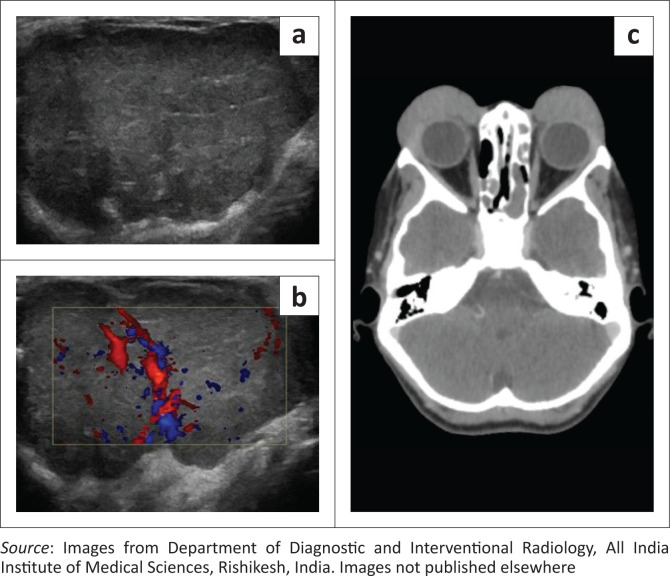
Grey scale (a) and Colour Doppler (b) images through the eyelid show a hypoechoic soft tissue mass in the eyelid with increased internal vascularity. (c) Axial Contrast Enhanced CT of the brain and orbits shows homogeneously enhancing soft tissue masses in both preseptal regions.

Initial investigation was with ultrasonography (USG) of the cheek and neck (GE Logiq S8, USA), which revealed diffuse enlargement of both parotid and submandibular glands. Multiple enlarged hypoechoic lymph nodes with a lobulated outline, increased vascularity and loss of fatty hila were noted at the parotid, submental and submandibular stations bilaterally. On ultrasound elastography, the lymph nodes showed an intermediate elasticity score (Figure 1). Ocular USG showed hypoechoic soft tissue with marked internal vascularity in both upper eyelids in the preseptal regions (Figure 2a, b). Both the eye globes were unremarkable. Her chest radiograph appeared normal.

**FIGURE 3:**
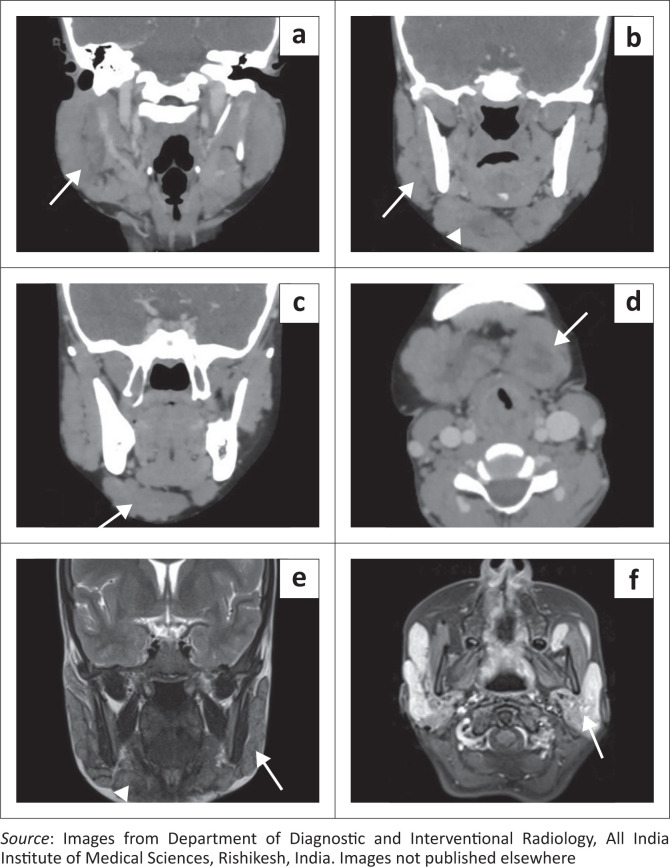
Coronal and Axial contrast enhanced CT images of the face show enlarged parotid (a: arrow), submandibular glands (b: arrowhead) and multiple enlarged lymph nodes at the parotid (b: arrow), submental (c: arrow) and submandibular stations (d: arrow). (e) Coronal T2WI MRI image shows hypointense signal of the lymph nodes (arrow and arrowhead). (f) Axial post contrast T1W MRI image shows intense homogeneous post-contrast enhancement of the parotid gland (arrow) and lymph nodes.

A contrast-enhanced (CE) CT scan of the face and neck (Philips Ingenuity Core 64 slice CT) was performed. Homogeneously enhancing soft tissue masses were seen in the preseptal regions of the eyelids (Figure 2c). Multiple enlarged homogeneously enhancing bilateral cervical and parotid lymph nodes were seen (Figure 3a–d).

**FIGURE 4:**
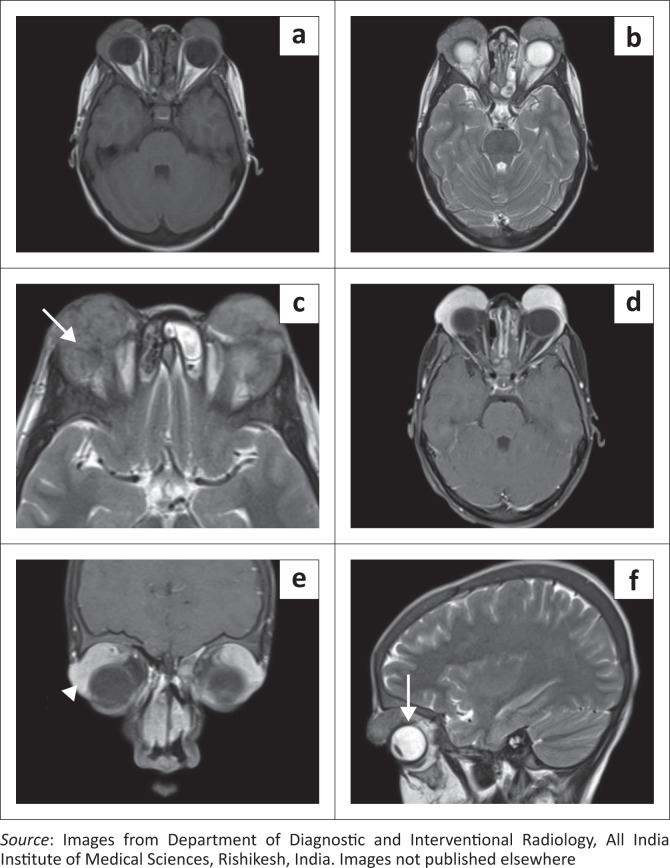
Axial T1WI (a), T2WI (b, c) and post-contrast T1WI (d) images show T1WI/T2WI hypointense soft tissue masses in the preseptal regions of both eyelids showing intra-orbital extension in the extraconal space (c: arrow) and intense homogeneous post-contrast enhancement (d). (e) Coronal post-contrast T1WI image shows intense enhancement of the lacrimal glands bilaterally (arrowhead). (f) Sagittal T2WI image shows that the extraconal soft tissue mass is closely abutting the superior rectus muscle (arrow).

Assessing for the disease extent in the face and orbit and for any intracranial involvement, contrast enhanced MRI of the face, orbit and brain (Siemens Magnetom Aera 1.5 Tesla MRI, Germany) was acquired. The enlarged lymph nodes and salivary glands showed T2W hypointense signal and homogeneous post-contrast enhancement (Figure 3e, f). Homogeneously enhancing soft tissue masses were seen in the preseptal regions of the upper eyelids. The masses were hypointense on T1-weighted (T1WI) and T2-weighted (T2WI) sequences. No diffusion restriction was seen. There was extension into the extraconal compartment of the orbits, with abutment of the superior and lateral recti bilaterally. No intraconal or intracranial extension was seen. Both lacrimal glands were bulky showing T2W hypointense signal and homogeneous post-contrast enhancement (Figure 4).

**FIGURE 5:**
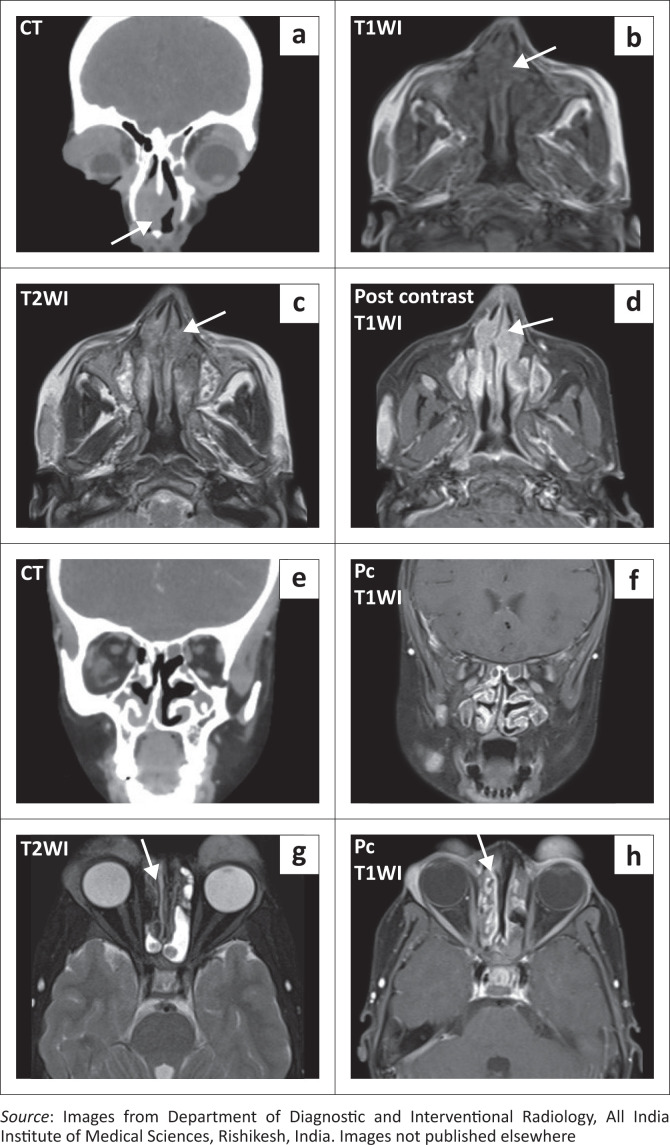
(a) Coronal Contrast Enhanced Computed Tomography (CECT) image of the face shows an enhancing soft tissue mass along the nasal septum (arrow). (b-d) Axial MRI images show intensely enhancing T1WI/T2WI hypointense nodular thickening in the nasal cavity (arrows) and bilateral maxillary sinuses. (e-f) Coronal CECT and Post-contrast T1WI MRI images show sinusitis and enhancing mucosal thickening along the paranasal sinuses and nasal turbinates. (g-h) Axial MRI images show sinusitis and nodular thickening. Note: The T2WI low signal nodular thickening shows intense post-contrast enhancement (arrows).

Pansinusitis was noted with homogeneously enhancing nodular thickening seen in the frontal, ethmoidal and maxillary sinuses bilaterally, along the nasal septum and involving the nasal turbinates. The corresponding CT scan showed no erosions of the sinus walls (Figure 5).

**FIGURE 6:**
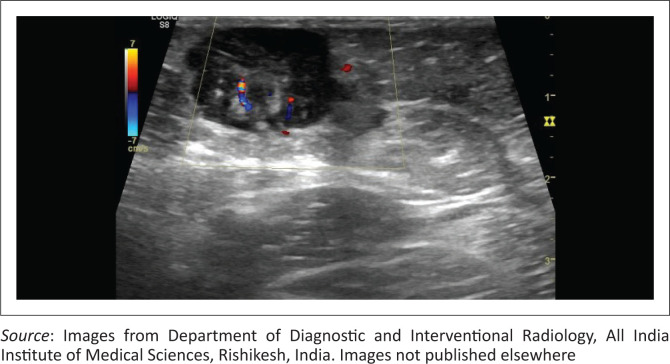
Colour doppler image of the right thigh shows an oval, solid soft tissue lesion in the subcutaneous plane. The lesion shows increased vascularity on colour doppler. It is focally compressing the underlying muscles.

Focused ultrasound of the right thigh swelling revealed a hypoechoic soft tissue lesion in the subcutaneous plane with mild to moderate internal vascularity on colour doppler (Figure 6). Abdominal ultrasound examination showed multiple sub-centimetre retroperitoneal and mesenteric lymph nodes.

**FIGURE 7:**
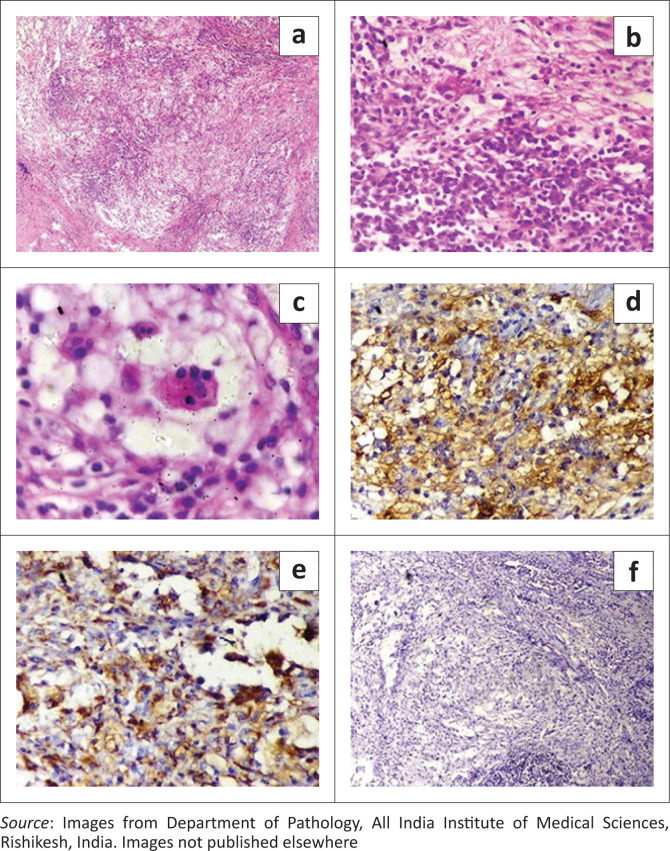
Pictomicrographs (H&E) show (a) replacement of normal lymph nodal tissue by histiocytes (100X), (b) histiocytes with abundant eosinophilic cytoplasm and (c) vesicular nucleus (400X), and (d) Emperipolesis. (d–f) Immunohistochemistry images show that the histocytes are S100 (d), CD68 (e) positive (DAB-400X) and CD1a (f) negative (DAB-100X).

The patient underwent an excision biopsy of the submental lymph nodes under general anaesthesia. Histopathological examination showed multiple partially encapsulated lymph nodes with loss of normal architecture and markedly widened sinuses. The sinuses of the lymph nodes were distended with lymphocytes, plasma cells and histiocytes. Marked ‘emperipolesis’ was seen. The histiocytes were positive for CD68 and S100 and negative for CD1a (Figure 7). These pathological features led to a definitive diagnosis of RDD. Osseous involvement was excluded by an unremarkable bone scan.

The main imaging differential diagnosis included lymphoproliferative disorders like lymphoma and infection. Lack of restricted diffusion and lack of organomegaly were not in favour of lymphoma. Lack of fever, local tenderness, normal leukocyte count and non-necrotic lymph nodes made the possibility of infection less likely. The long period of evolution of the pathological process also favoured the benign nature of the disease rather than lymphoma or infection.

## Management and outcome

The benign and indolent course of the disease was explained to the patient. She was started on prednisolone therapy and was followed up regularly. There was no need for surgery given the lack of any significant complications. Follow-up after 6 months showed only a mild reduction in the size of the parotid and cervical lymph nodes. No significant increase or decrease was noted in the size of eyelid masses or subcutaneous thigh swelling. However, because of associated side effects and reduced serum calcium levels, the steroids were discontinued. After explaining all the risks and benefits of chemotherapy in RDD, the patient’s family refused consent for a trial of chemotherapy. The patient was further advised regular follow-up and was asked to report immediately in case of any suspected complications.

## Discussion

Rosai Dorfman disease is a well-established but rare disease entity that involves the proliferation of histiocytes. It was first described by Rosai and Dorfman in 1969 and was originally termed Sinus Histiocytosis with massive lymphadenopathy (SHML).^2,3^ It is also known as Rosai Dorfman Destombes disease. The term RDD is now preferred as few cases may present solely as extranodal disease.^2^ The paranasal sinuses (PNS) are a common site of involvement but the term sinus histiocytosis, as described originally, refers to histiocytic infiltration of the medullary sinus of lymph nodes.^4^

The aetiopathogenesis of RDD is not known. Immune-mediated dysfunction is one of the proposed theories. Other causes may include infectious or neoplastic processes.^5^ Some authors have mentioned it as a disorder of the mononuclear phagocyte and immunoregulatory effort (M-PIRE) system. Association with Ebstein-Barr virus was also sought^6^; however, no single cause has been validated.

Children and adolescents are predominantly affected by the disease with 80% of patients being less than 20 years of age. Isolated extranodal disease is more common in the elderly population.^6^ A slight male preponderance is seen with a greater incidence of the disease in African and Western Indian populations.^1,4^

Painless cervical lymphadenopathy is the most common presentation. There may be associated fever, leukocytosis, raised ESR and hypergammaglobulinaemia.^2,4,7^ Other lymph nodes, including the axillary, mediastinal, retroperitoneal and inguinal regions, can also be involved.^1^ The lymph nodes may become very large in size and cause mass effect on vital structures. The ultrasound features of the involved lymph nodes can appear similar to malignant infiltration.^4,8^ However, no case of malignant transformation of RDD has been reported in the literature.^4^ The involved lymph nodes have been shown to be Gallium and^18^ FDG avid.^4^

Extranodal involvement has been reported in approximately 43% of patients^2,9^ while isolated extranodal disease without nodal involvement has been reported in approximately 23% of cases.^2^ Any organ can be affected by RDD.^1^ The head and neck is the most common extranodal region with the commonest site being the skin, followed by the nasal cavity and PNS, subcutaneous tissue, orbits, eyelids and bones.^4^ Extranodal head and neck involvement tends to occur more frequently in patients with immunological disturbances.^4,10^

Nose and PNS involvement can present as nasal obstruction, epistaxis or olfactory abnormalities. Cross-sectional imaging can show enhancing polypoidal masses, thickening of the mucosa or opacification of the involved sinuses or nasal cavity. The lesions are usually hypointense on T2-weighted MRI. Bone erosion and adjacent soft tissue involvement are usually not seen. This in contrast to destructive diseases like granulomatosis with polyangiitis and lymphoproliferative disorders that form close differentials of the nose and PNS involvement.^4^ The PNS involvement in the presented case also had similar imaging features with enhancing T2WI hypointense polypoidal thickening and pansinusitis but no osseous erosion. Salivary gland lymphoid hyperplasia seen in the current case is another common presenting feature of RDD.^1^

Involvement of the orbit and eyelid can be seen in 7% – 10% of cases of RDD.^4^ It can be isolated or may be associated with skin or lymph node disease.^11^ Unlike the presented case, unilateral involvement of the orbit is more common. Patients can present with proptosis, limitation in the range of eye movements, swelling of the eyelids or decreased vision.^2^ An extraconal variably enhancing soft tissue mass with infiltration of the surrounding structures is the commonest manifestation on imaging.^1,2,4^ Involvement of the eyelids can be in the form of large preseptal (as in this case) or postseptal masses.^2^ Involvement of the lacrimal gland in RDD is a known entity and was seen in this case as bulky enhancing lacrimal glands. Involvement of the eye globe can also be rarely seen. Several cases of intracranial extension of the orbital masses have also been reported in RDD, though this is rare.^1^ A myriad of diseases that can mimic orbital involvement of RDD include lymphoma, orbital pseudotumor, sarcoidosis, haemangioma or lymphatic malformation like lymphangioma.^2^

Intracranial involvement in RDD is a rare but known manifestation. Typically, extra axial dural-based enhancing masses are seen, which have a characteristic ‘markedly T2WI hypointense’ signal on MRI. This feature helps to distinguish it from meningioma, which is typically T2WI hyperintense, unless calcified.^1,2,4,12^ Another feature of meningioma, which is lacking in RDD, is hypervascularity on angiography. About 70% of RDD patients with intracranial involvement have no associated lymphadenopathy.^4^

Osseous involvement is rare (<10%) in RDD but usually occurs in association with nodal disease. Isolated osseous involvement is reported in the literature but is extremely rare.^7^ Multicentric lytic lesions within the medulla of long bones like the tibia or fibula or lytic skull lesions are the typical imaging features on radiographs and CT. Langerhans cell histiocytosis (LCH) is the main imaging differential of osseous involvement in RDD.^1^ One of the differentiating features is the ill-defined fuzzy margins of the lytic lesions in the healing phase of RDD rather than well-defined sclerotic margins seen in LCH.^4^

Subcutaneous involvement is known and was seen in this case as a subcutaneous thigh lesion. Thoracic involvement has been reported in a few cases. These cases included RDD presenting as a lung mass in one report and epicardial involvement in another case.^1,13^

The imaging manifestations of RDD (summarised in Table 1) are variable, and nonspecific and histopathology is necessary for a definitive diagnosis. Rosai Dorfman disease is a histiocytic disorder with an abundance of histiocytes. The classic pathological feature termed ‘Emperipolesis’ refers to phagocytosis of lymphocytes, polymorphonuclear leukocytes, plasma cells or erythrocytes within the cytoplasm of the histiocytes. On immunohistochemistry, the histiocytes are positive for S100 and CD68 and negative for CD1a.^1,10^ All these features were seen in the presented case and helped clinch the final diagnosis.

**TABLE 1:** Imaging features of Rosai Dorfman disease.

Location	Site or organ	Imaging manifestations	Differential diagnosis
**Nodal involvement**	Cervical: Most common	Painless cervical lymphadenopathy: Most common presentation	Lymphoproliferative disorders
		Can become very large and cause mass effect	
		Indistinguishable from malignant lymphadenopathy	
		Gallium and 18 FDG Avid	
	Others (In descending order of involvement): Inguinal Axillary Mediastinal Retroperitoneal	May also be involved	
**Extranodal involvement**			
Head and neck	Nose and paranasal sinuses	Enhancing polypoidal masses, Mucosal thickening, Opacification of sinuses and/or nasal cavity	Granulomatosis with Polyangiitis; Lymphoproliferative disorders
		Hypointense on T2-weighted imaging (T2WI)	
		Erosions of bones and soft tissues: Usually not seen	
	Orbit	Unilateral involvement more common	Lymphoma, Orbital Pseudotumor, Sarcoidosis, Hemangioma
		Extraconal soft tissue mass showing infiltration into the surrounding structures; Hypointense on T2WI; Variable enhancement	
		Lacrimal gland involvement: Increase in the size with post-contrast enhancement	
		Eye globe involvement: Rare	
	Eyelids	Large preseptal or postseptal masses	
	Salivary glands	Lymphoid hyperplasia: common, seen as enlarged glands with post-contrast enhancement	Lymphoproliferative disorders
Skin and Subcutaneous Tissue	Skin	Commonest site of involvement in head and neck	
	Subcutaneous Tissue	Soft tissue masses	
Central nervous system	Intracranial and spinal disease	Rare	Meningioma
		Extra axial dural-based enhancing masses: Characteristic ‘Markedly T2WI hypointense’ signal, Lack of hypervascularity on angiography	
Bones (5% – 10%)		Rare involvement (<10%)	Langerhans cell histiocytosis
		Usually associated with nodal disease	
		Multicentric lytic lesions within the medulla of long bones or lytic skull lesions	
Thorax (4%)	Lung	Rarely involved. Reported cases showed:	
		Reticulonodular thickening	
		Lung nodules	
		Lung mass in a case report	
	Breast	Rare involvement. Can present as:	
		On mammogram: mass, architectural distortion, focal asymmetry	
		On ultrasound: Hypoechoic mass showing angulated margins and vascularity on colour Doppler	
	Heart	Rare reported cases of infiltrative intracardiac mass; epicardial involvement	

Rosai Dorfman disease is a benign disorder with an indolent course, good prognosis and often does not require any therapy. Treatment is needed in cases showing involvement or compression of vital organs or the ones causing life-threatening complications. Therapy in such cases is limited to surgical resection and/or debulking. Radiation and steroid therapy have been tried with variable results. Chemotherapy is considered largely ineffective.^1,14^

## Conclusion

This case was a typical case of RDD affecting an adolescent female with nodal and extranodal involvement. However, lymphoma was still the main differential on imaging. This highlights the rarity of the disease that is often missed in the differential diagnosis even in cases with classic features. Knowledge of the disorder can help guide optimal management and avoid unnecessary treatment in this self-limiting disease. Histopathology remains the gold standard and is necessary for a definitive diagnosis.
